# Integrated transcriptomics and metabolomics analysis reveals key regulatory network that response to cold stress in common Bean (*Phaseolus vulgaris* L.)

**DOI:** 10.1186/s12870-023-04094-1

**Published:** 2023-02-09

**Authors:** Xiaoxu Yang, Chang Liu, Mengdi Li, Yanmei Li, Zhishan Yan, Guojun Feng, Dajun Liu

**Affiliations:** 1 Horticulture Department, College of Advanced Agriculture and Ecological Environment, Heilongjiang University, Harbin, 150000 China; 2 Horticulture Department, College of Life Sciences, Heilongjiang University, Harbin, 150000 China

**Keywords:** Common bean, Cold stress, Transcriptomics, Metabolomics, Regulatory network

## Abstract

Cold temperatures can be detrimental to crop survival and productivity. Breeding progress can be improved by understanding the molecular basis of low temperature tolerance. We investigated the key routes and critical metabolites related to low temperature resistance in cold-tolerant and -sensitive common bean cultivars 120 and 093, respectively. Many potential genes and metabolites implicated in major metabolic pathways during the chilling stress response were identified through transcriptomics and metabolomics research. Under chilling stress, the expression of many genes involved in lipid, amino acid, and flavonoid metabolism, as well as metabolite accumulation increased in the two bean types. Malondialdehyde (MDA) content was lower in 120 than in 093. Regarding amino acid metabolism, 120 had a higher concentration of acidic amino acids than 093, whereas 093 had a higher concentration of basic amino acids. Methionine accumulation was clearly higher in 120 than in 093. In addition, 120 had a higher concentration of many types of flavonoids than 093. Flavonoids, methionine and malondialdehyde could be used as biomarkers of plant chilling injury. Transcriptome analysis of hormone metabolism revealed considerably greater, expression of abscisic acid (ABA), gibberellin (GA), and jasmonic acid (JA) in 093 than in 120 during chilling stress, indicating that hormone regulation modes in 093 and 120 were different. Thus, chilling stress tolerance is different between 093 and 120 possibly due to transcriptional and metabolic regulation.

## Backgrounds

Suboptimsl low temperature affects plants throughout their life cycle, thereby disturbing their growth and development and serving as a typical limiting factor in agricultural productivity [1]. Low temperatures cause worldwide agricultural losses, and chilling stress and freezing stress are two types of low-temperature impacts on plants. Leaves wilt and physiological processes are hindered in plants under cold stress. During the course of evolution, plants have developed various methods to cope with low temperature stress, including changes in morphological, physiological, biochemical, and molecular processes [2–5], such as gene expression, enzyme activity, and metabolic stability. Plants mitigate the impact of cold stress through sophisticated gene regulatory networks and highly controlled metabolic networks [6]. Cold treatment induces many biochemical pathways, including glucose metabolism [7] and amino acid metabolism [8], which are important for the synthesis of not only proteins but also precursors of various metabolites and metabolic products with multiple functions in plant responses to cold. Furthermore, studies have revealed that the regulation of plant hormones, including abscisic acid (ABA) [9, 10], gibberellic acid (GA) [11], and jasmonic acid (JA) [12], changes during cold stress.

Plants on exposure to low temperature acquire freezing tolerance. Freezing injury in most plants and tissues occur by from severe cellular dehydration that occurs upon ice formation and cellular membrane systems are a primary site of freeze-induced injury. Cold responsive genes get activated in response to low temperature. Malondialdehyde (MDA) is an important product of cell membrane peroxidation, and its content reflects the extent of cell membrane destruction. MDA is usually used as an indicator of membrane lipid peroxidation. Low temperature causes the increase of MDA content, which has been confirmed in cucumber [13], tomato [14] and other crop seedlings.

Cold response is a highly complex process that involves physiological and biochemical modifications. Furthermore, alterations of the expression patterns of many genes, proteins and metabolites in response to cold stress have been reported. Plants require specialized mechanisms to survive exposure to low temperature. Cold stress can be classified as chilling (0– 15 °C) and freezing (< 0 °C) stresses.. Multiple primary sensors are thought to be involved in stress sensing. Each sensor may perceive a specific aspect of the stress and may be involved in a distinct branch of the cold signaling pathway. Plants may sense low temperature through changes in the physical properties of membranes, because membrane fluidity is reduced during cold stress [15]. Low temperature or cold stress is one of the major abiotic stresses for bean seeds production. Bulgari analyzed the effeures like room temperature, at 3° C and at—19° C. Seeds were germinated in germination box. Germination percentage, rate, seed vigour and germination index was measured. Respiration response was also analyzed. The procedure does not quantify a respiration rate, but it indicates that respiration rate can be compared across different treatments. Observation from conductivity measurement shows that leachate solution of -19° C gives low conductivity whereas 3° C gives moderate and at normal room temperature it was high. Low temperature showed high amylase as well as catalase activity. Room temperature showed a good response against few parameters but not in enzyme activity [16]. Studies have shown that Galactinol synthase (GolS) is considered to be a key regulator of the biosynthesis of Raffinose family oligosaccharides (RFOs). Accumulation of RFOs has been reported to play a role in protection against abiotic stresses. Etsuo and Kenjirou identified two cDNAs encoding galactinol synthase from wheat (*Triticum aestivum* L.), which designated as TaGolS1 and TaGolS2. Expression of the two TaGolS genes was induced by cold stress but not by drought, heat stress or ABA treatment in wheat. They generated transgenic lines of rice (Oryza sativa L.) constitutively overexpressing TaGolS1 or TaGolS2. These transgenic plants accumulated significantly higher levels of galactinol and raffinose than did wild-type plants and exhibited enhanced cold-stress tolerance. The results demonstrate the involvement of galactinol and raffinose in the development of chilling stress in rice and indicate that the genetic modification of the biosynthesis of RFOs by transformation with GolS genes could be an effective method for enhancing chilling-stress tolerance in rice [17].

Transcriptomics is the study of all transcripts, which are the fundamental connection between genes and functions, produced in tissues and cells under specific circumstances. Transcriptome sequencing technology has a high degree of accuracy and coverage. It offers a sample’s sequence structure and more effectively conveys regulatory information at the transcriptome level. This technology is currently applied extensively in health, agriculture, and other domains. Agronomy research more commonly involves studies related to mechanisms underlying stress-induced plant adversity, which includes abiotic (drought, water, temperature, and salt) and biotic stress (Fusarium oxysporum, cyst nematode infection, and SMV) [18]. The regulatory mechanism of plant stress response is extremely complex, and transcriptome studies alone do not presnt the complete picture of the various mechanisms and their interactions that lead to improved cold stress. In order to get much deeper insights into the mechanisms that are implicated in cold stress response, transcriptomics can be integraed with metabolomics. Metabolomics research is based on identifying the type and quantity of metabolites in an organism, which may be directly linked to the organism’s genes and phenotypes, to better understand its regulatory system [19]. Metabonomics is mostly used to extract differential metabolites and study metabolic pathways or networks. As an emerging science, metabonomics has been widely used in pathology and pharmacology and to study the response mechanism of metabolites produced after stress. Because of the continual advancement of bioinformatic tools, metabolomics is extensively used to study the properties of plant metabolites produced under various stresses [20]. Using the combination of transcriptome and metabolome analyses, previous studies have investigated the impact of cold stress on transcriptome and metabolome profiles in crops like tobacco [21], wheat [22], rape [23], and other plant species. In common bean, transcriptome analysis has been used to examine gene expression patterns under salt [24] and drought stress [25]. However, no study has been conducted integrated transcriptomics and metabolomics analysis of common bean under cold stress. Common bean is a pod seed and food crop with high nutritional value [26] and a large planting area worldwide. Chilling injury (0 °C–15 °C) severely limits bean production, especially in low latitudes. Chilling can damage different growth stages, thus significantly affecting grain yield and quality of bean. Low temperature can also suppress seed germination. At the seedling stage, sudden exposure to low temperature can cause injury to leaves, and in extreme cases, it can lead to seedling death. At the flowering and podding stages, low temperature can increase the falling of petals and bean pods, thereby severely reducing production.

Through variety screening, we found that 093 is more susceptible to low temperatures than 120. Consequently, we used transcriptomics and metabolomics to define the response of the two ecotype common beans to low temperatures, as well as investigate their distinct chilling stress mechanisms. The effects of chilling stress on transcription and metabolism levels in the two bean types were explored to learn more about their cold tolerance mechanism. Our findings imply that cold domestication is linked to considerable alterations in the transcriptome and metabolome, but that minor differences in sensitivity to chilling stress may influence the sensitivity of both cultivars. Between 093 and 120, genes and metabolites induced due to cold response were comparable, but variations were noted in photosynthesis, hormones, lipids, and flavonoids.

## Materials and methods

### Plant material and cold treatment

The cold-sensitive common bean variety 093 and cold-tolerant common bean variety 120, provided by the Horticulture and Seed Research Group of Heilongjiang University, were used in this study. 093 and 120 are the germplasm resources collected by our research group, and they are not related in pedigree. The control groups under non-chilling stress were 093CK and 120CK, and the treatment groups under chilling stress were 093 T and 120 T. The seeds were initially planted in a pot with a 10-cm inner diameter, and six pots were maintained for each variety, with three seeds per pot. To eliminate the impact of major environmental factors, the seeds were cultivated in climate chambers at 25 °C 12-h light/12-h dark with 300 μmol m − 2 s − 1 light. The plants were grown to the trifoliate leaves emerged and exposed to 4 °C low temperature. There were three replicates per treatment and ten plants per replicate. Then, photosynthetic indices were measured using a photosynthetic apparatus (Li-6400. After 3 days of treatment, leaves at the same position on the plants in the treatment and control groups (25 °C) were flash-frozen in liquid nitrogen and stored at − 80 °C prior to being used in the experiments. Total RNA was extracted from the leaf tissue using TRIzol reagent (Invitrogen) according to the manufacturer’s instructions.

### Determination of malondialdehyde content

A small amount of plant tissue (leaf) was taken, the mass was denoted as W, 5 mL of 5%TAC (trichloroacetic acid) was added, and the homogenate obtained after grinding was centrifuged at 3000 r/min for 10 min. Then slowly absorb 2 ml of supernatant with a pipette gun, add 2 mL of 0.67%TBA (thiobarbituric acid), mix, boil for 15 min in a water bath at 100℃, and centrifugal once (10 min) after cooling. The absorbance values of supernatant at 450 nm, 532 nm and 600 nm were determined respectively, using 2 mL distilled water plus 2 mL 0.67%TBA as control. According to the following formula:

$$\mathrm{MDA}\;\left(\mathrm\mu\mathrm m\mathrm o\mathrm l/\mathrm g\;\mathrm{FW}\right)\;=\;\left(\mathrm C\times\mathrm V\right)/\mathrm W$$

$$\mathrm C\;\left(\mathrm\mu\mathrm m\mathrm o\mathrm l/\mathrm L\right)\;=\;6.45\times\left(\mathrm D532\;-\;\mathrm D600\right)\;-\;0.56\times\mathrm D450$$

C represents MDA concentration, and D450, D532, and D600 represent the absorption values at wavelengths of 450, 532, and 600 nm, respectively.

### RNA isolation and library preparation for transcriptome analysis

The TRIzol kit was used to extract total RNA from leaves (TaKaRa, Dalian, China). The manufacturer’s instructions were followed to determine the quantity and quality of total RNA (Agilent 2100 Bioanalyzer). The entire RNA was subsequently submitted to LC Biotech in Hangzhou, China, for building RNA-Seq library and RNA sequencing. For paired-end sequencing, an Illumina HiSeq2000 sequencer was employed (29,100 bp).

### Transcriptome flow

The leaf tissue exposed to chilling stress was quickly frozen with liquid nitrogen. Three biological replicates were used in the RNA-Seq experiment. The MiniBEST plant RNA extraction kit was used to extract total RNA from the plants. The Illumina Hi-SEQ2500/Illumina NextSeq500 platform was used to sequence all samples. An internal Perl script (https://www.perl.org/) was initially used to process raw data in FASTQ format. Reads containing the adaptor and ploy-n, and low-quality reads were eliminated from the raw data at this stage, producing clean data. The Phytozome bean database (https://phytozome.jgi.doe.gov/pz/ portal.html) was used to align sequences.

### Metabolome flow

Frozen leaf tissue (1 g) was ground in liquid nitrogen and thawed on ice. The metabolite analysis was performed in six biological replicates. Metabolites were extracted with 50% methanol buffer. After being thawed, the samples were precooled with 120 μL of 50% methanol, extracted with 20 μL, vortexed for 1 min, and incubated at room temperature for 10 min. The extracted mixture was stored at − 20 °C overnight. After centrifugation at 4000 g for 20 min, the supernatant was transferred to a new 96-well plate. Quintuple metabolite accumulation was measured using completely independent tissue samples. Each extract was combined into 10 μL to form a mixture. All samples were collected using the liquid chromatography mass spectrometry (LC–MS) system, according to the instructions on the machine (www.lc-bio.com).

First, an ultra-high performance liquid chromatography (UPLC) system (Sciex, UK) was used for chromatographic separation. Reversed-phase separation was performed on the Acquity UPLC T3 column (100 mm*2.1 mm, 1.8 μm, Waters, UK). The column oven was maintained at 35 °C. The flow rate was 0.4 mL/min. The mobile phase consisted of solvent A (water, 0.1% formic acid) and solvent B (acetonitrile, 0.1% formic acid). Gradient elution conditions set were as follows: 0–0.5 min, 5% B; 0.5–7 min, 5%–100% B; 7–8 min, 100% B; 8–8.1 min, 100%–5% B; 8.1–10 min, 5% B; 4 μL for each sample.

Metabolites eluted from the column were detected using a high-resolution tandem mass spectrometer Triple TOF 5600 plus (Sciex, UK). Online KEGG (http://www.kegg.jp/) and HMDB (http://www.hmdb.ca/) databases were used to annotate the metabolites in the database with the accurate molecular weight data (M/Z) of matched samples. If the mass difference between the observed and database values was < 10 ppm, the metabolite was annotated and further confirmed by the molecular formula isotope distribution measurements. We also validated the identified metabolites using an internal fragment spectrum library of metabolites. Outlier detection and effect evaluation were performed using the pretreated data set.

### Differentially Expressed Genes (DEGs) were verified by real-time fluorescence quantification

For verification, real-time fluorescence quantitative PCR (RT-QPCR) was used to randomly select eight genes from among the chilling stress response genes identified in the RNA-Seq experiment. Oligo 7.0 (ver.) was used to design gene-specific primers. Quantitative real-time PCR was performed using Roche Molecular Systems Inc. (Branchburg, NJ, USA). The 20-μL reaction system consisted of 2.5 Real MasterMix (9 μL), 1 μL forward and reverse primers, and 4 μL lcDNA. Relative gene expression was quantified using the 2-ΔΔCq method, with ACTION as the internal reference gene.

### MapMan analysis

Based on the results of transcriptome and metabolome, MapMan 3.5.1 (http://mapman.gabipd.org/web/guest/mapman) was used to analyze the DEGs whose differential expression multiples were more than 4 times. Using this software, functional grouping and metabolic pathway mapping were performed.

## Results

### Effects of chilling stress on photosynthetic indices and mda content of common Bean (Phaseolus vulgaris L.)

**Fig. 1:**
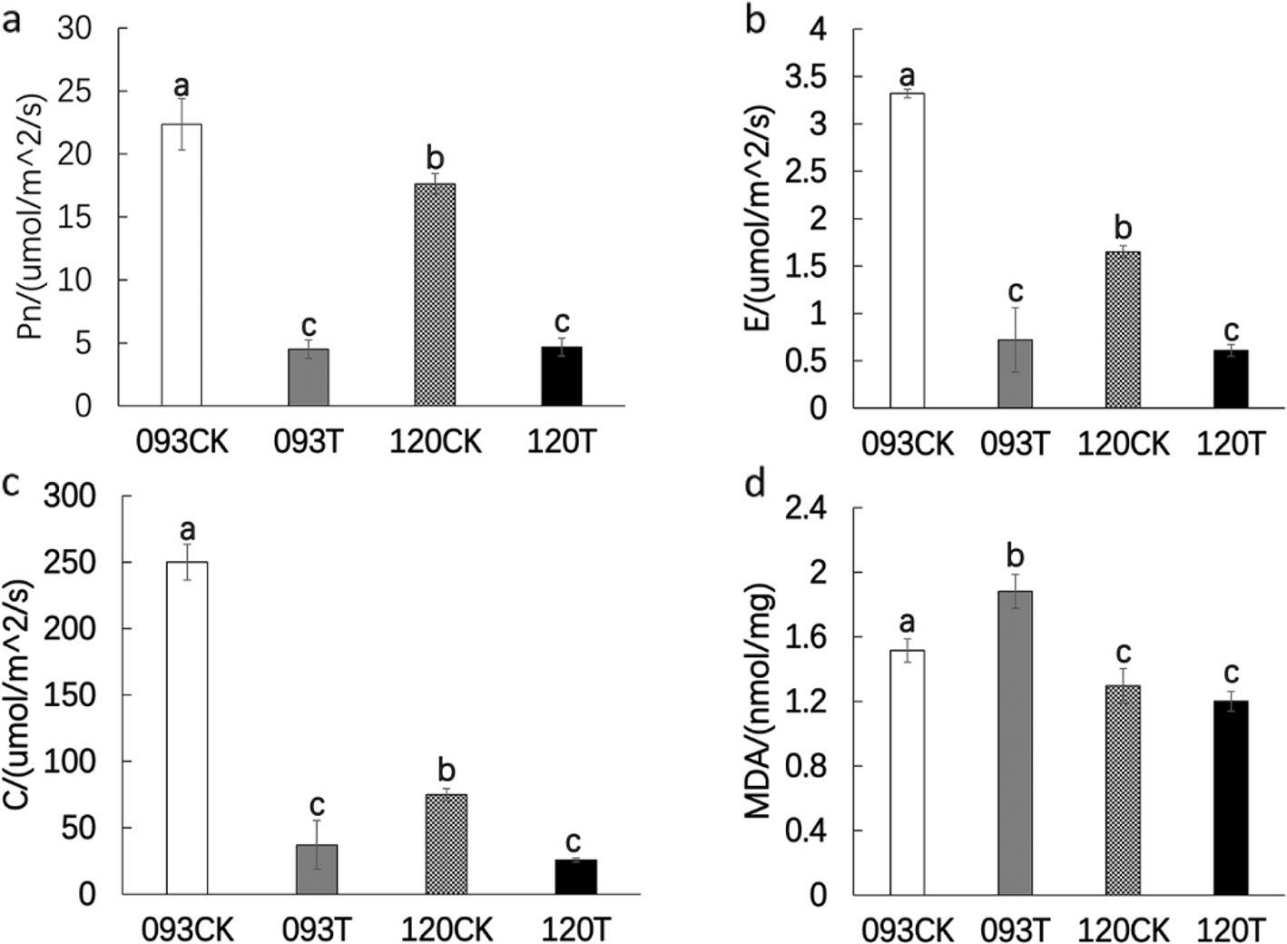
Changes in photosynthetic indices and MDA content of 093 and 120 under chilling stress. **a** Net photosynthetic rate measured using a photosynthetic index meter; **b** transpiration rate measured using a photosynthetic index meter; **c** stomatal conductance measured using a photosynthetic index meter; **d** MDA content in leaves of 093 and 120 under chilling stress

To investigate the impact of chilling stress on photosynthetic indices of bean seedlings, we measured the net photosynthetic rate (Pn), transpiration rate (E), and stomata conductance (C) in 093 and 120 after 3days of chilling stress. In 093T, the Pn, E and C was signicantly lower compared with that of 093CK, and in120T, the Pn, E and C was also signicantly lower compared with that of 120CK. The MDA concentration in 093T increased dramatically after chilling stress, but that in 120T remained unchanged after chilling stress (Fig. 1). The degree of membrane damage in 120T was lower under chilling stress.

### Metabolome analysis of 093 and 120 under chilling stress

**Fig. 2:**
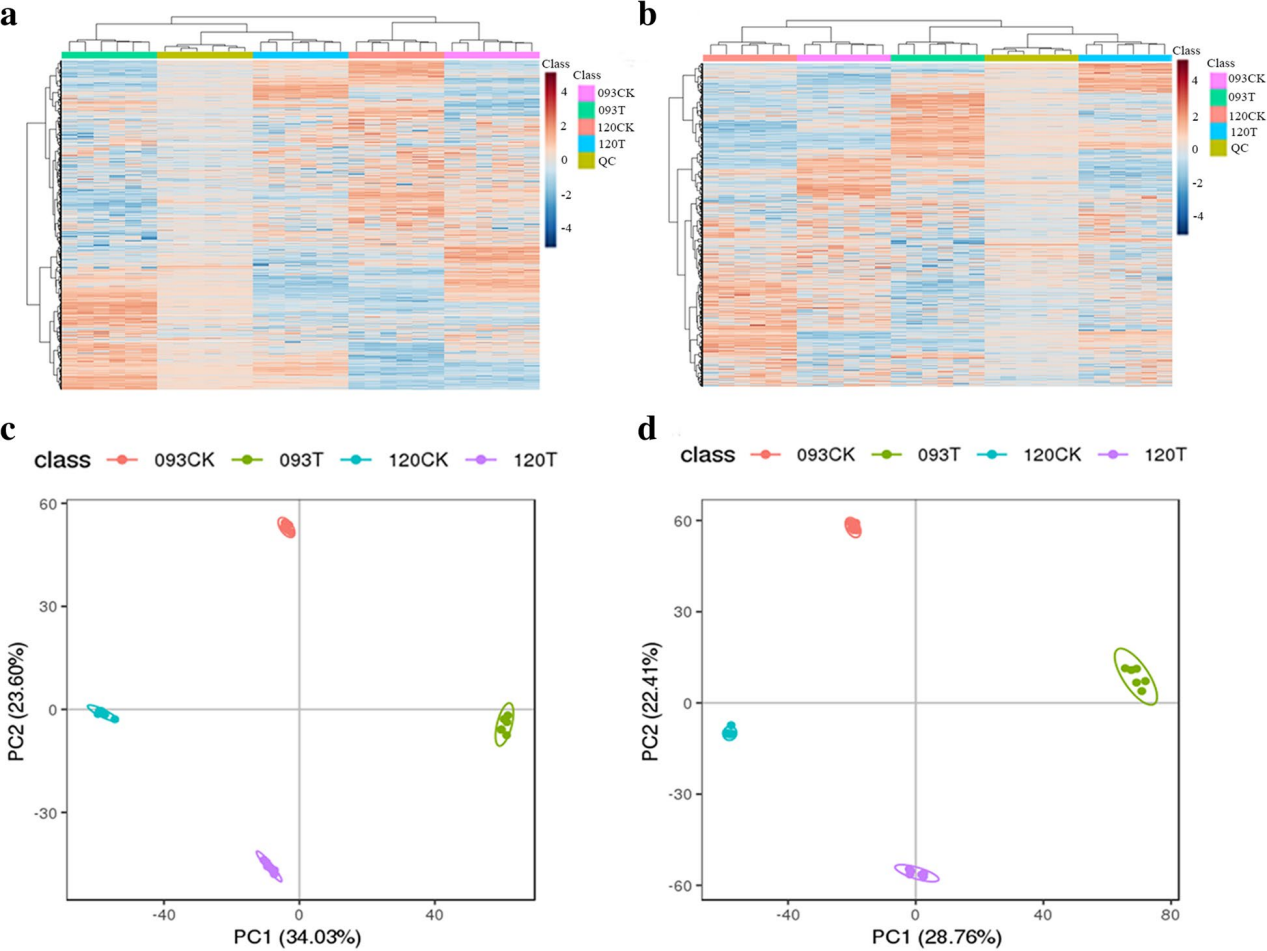
Cluster analysis results of metabolites detected in all samples. **a** is the metabolite detected under the positive ion mode, (**b**) is the metabolite detected under the negative ion mode. **c, d**, PCA results for all treatment groups. **c** is the metabolite detected under the positive ion mode, and (**d**) is the metabolite detected under the negative ion mode

A total of 18,166 metabolites were detected by LC–MS, and 7670 metabolites were annotated. Among them, 654 metabolites, including alkaloids and derivatives, benzenoids, lipids and functional-like molecules, and organic acids and derivatives, were significantly different. In the heat map (Fig. 2a and b), all biological replicates were grouped together, indicating a strong correlation among the replicates and high data reliability. The heat map also revealed that some metabolites only accumulated in 093, while some accumulated only in 120. PCA of metabolite data showed (Fig. 2c and d) that the biological repeats were closely projected in space, which also indicated a good correlation among the repeats.

### Transcriptomics analysis of 093 and 120 under chilling stress

**Fig. 3:**
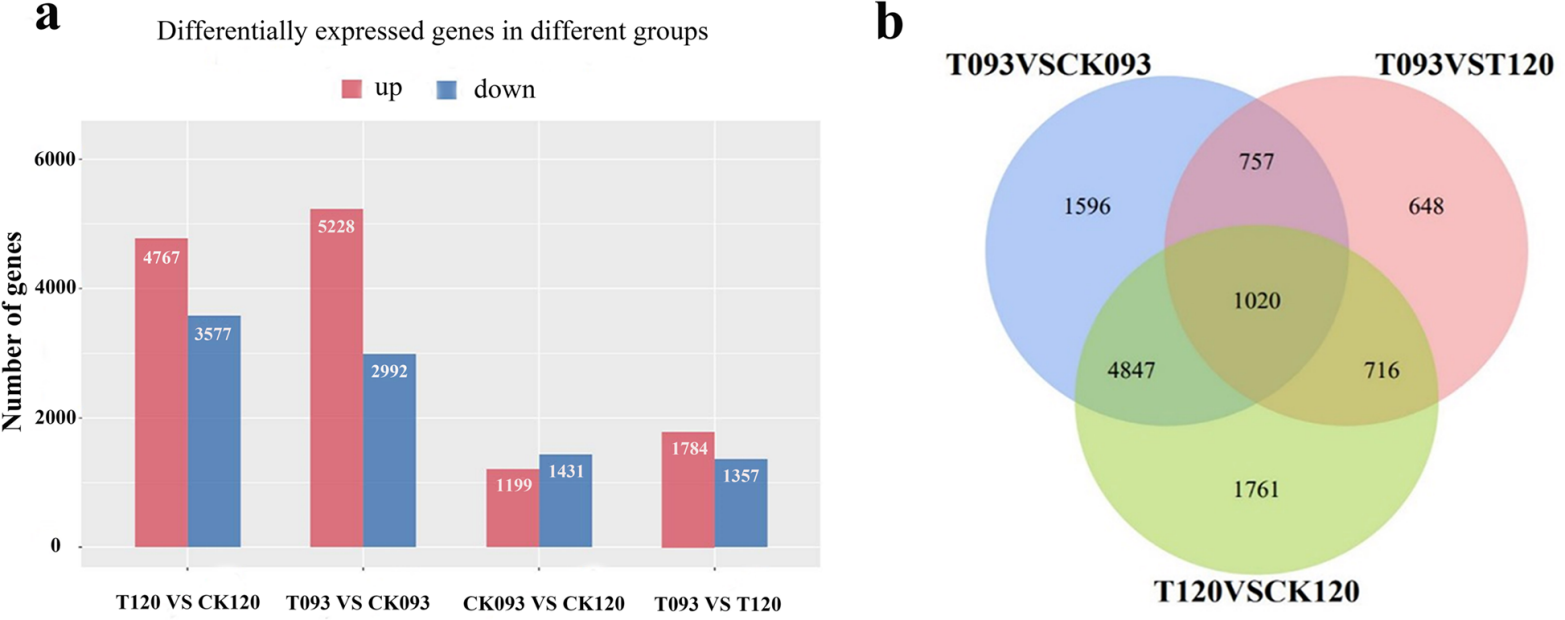
Number of differentially expressed genes. **a** shows the upregulation and downregulation of DEGs in different comparison groups and (**b**) shows the Venn plot of DEGs

Leaf tissue samples of seedlings from the 093C OR 093CK or 120C OR 120 CK (untreated control group) and 093 T or 120 T (low temperature stress group) groups were subjected to RNA-Seq. Twelve libraries were built and sequenced, allowing for three biological duplications of each sample and approximately 556 million raw reads. After removing cohesive sequences and low-quality data, a total of 531 million (95.57%) pure reads were retrieved, with an average of 44.25 million pure reads per sample. The common bean reference genome was found in 94.63% of the reads, and 64.39% of the reads were found in a single area (Tables 1 and 2). DEGs were determined according to the following criteria: absolute value of log2 ≥ 1 and q < 0.05. In the chilling stress-tolerant varieties (120Tvs120C) and cold-sensitive varieties (093Tvs093C), 12,978 upgrades and 9,357 DEG downgrades were observed. A total of 5228 and 4767 genes were upregulated, and 2992 and 3577 genes were downregulated in 093 and 120, respectively, compared with the control group. Moreover, 2630 and 3141 DEGs were identified in 093Cvs120C and 093Tvs120T (Fig. 3a), respectively. A Venn diagram showed that the intersection of 093Tvs093C and 120Tvs120C (representing the same number of DEGs in 093 and 120 after chilling stress) is 5867. In total, 1777 DEGs obtained from the intersection of 093Tvs120C and 093Tvs120T, representing significant cold response genes in 093. In total, 1716 DEGs obtained from the intersection of 120Tvs120C and 093Tvs120T represented significant cold response genes in 120 (Fig. 3b).

**Table 1:** Quality control of transcriptome data

Sample	Raw Data	Valid Data	Valid Bases	Valid Ratio (Reads)	Q20%
093T_1	37,369,116	36,697,286	5.50G	98.20	99.97
093T_2	50,774,510	50,055,538	7.51G	98.58	99.94
093T_3	40,722,272	39,636,566	5.95G	97.33	99.95
093CK_1	51,300,236	50,294,146	7.54G	98.04	99.81
093CK_2	50,821,910	48,843,450	7.33G	96.11	99.94
093CK_3	39,769,616	38,524,680	5.78G	96.87	99.97
120T_1	48,484,170	45,330,426	6.80G	93.50	99.95
120T_2	44,076,434	36,841,226	5.53G	83.58	99.90
120T_3	46,345,334	45,241,094	6.79G	97.62	99.95
120CK_1	47,207,050	46,442,518	6.97G	98.38	99.93
120CK_2	46,946,322	43,213,188	6.48G	92.05	99.92
120CK_3	51,818,472	49,919,852	7.49G	96.34	99.95

**Table 2:** Quality control of transcriptome data

Sample	Q30%	GC Content %	Mapped reads	Unique Mapped reads
093T_1	98.62	43.5	34,871,132(95.02%)	23,907,402(65.15%)
093T_2	98.04	44	47,325,029(94.55%)	31,694,576(63.32%)
093T_3	97.97	43.5	37,521,826(94.66%)	25,821,745(65.15%)
093CK_1	97.79	46.5	47,223,462(93.89%)	30,513,586(60.67%)
093CK_2	97.94	46.5	45,106,051(92.35%)	25,633,962(52.48%)
093CK_3	98.07	45	36,165,917(93.88%)	22,573,020(58.59%)
120T_1	97.97	43.5	43,280,338(95.48%)	31,671,342(69.87%)
120T_2	97.71	43.5	34,949,428(94.86%)	25,277,204(68.61%)
120T_3	97.98	44.5	42,875,663(94.77%)	29,412,270(65.01%)
120CK_1	97.94	44.5	44,125,274(95.01%)	30,135,693(64.89%)
120CK_2	97.99	44.5	41,285,687(95.54%)	29,737,219(68.82%)
120CK_3	98.17	44.5	47,709,936(95.57%)	35,005,882(70.12%)

### Comparison of metabolite and gene levels between 093 and 120 under the cold condition

**Fig. 4:**
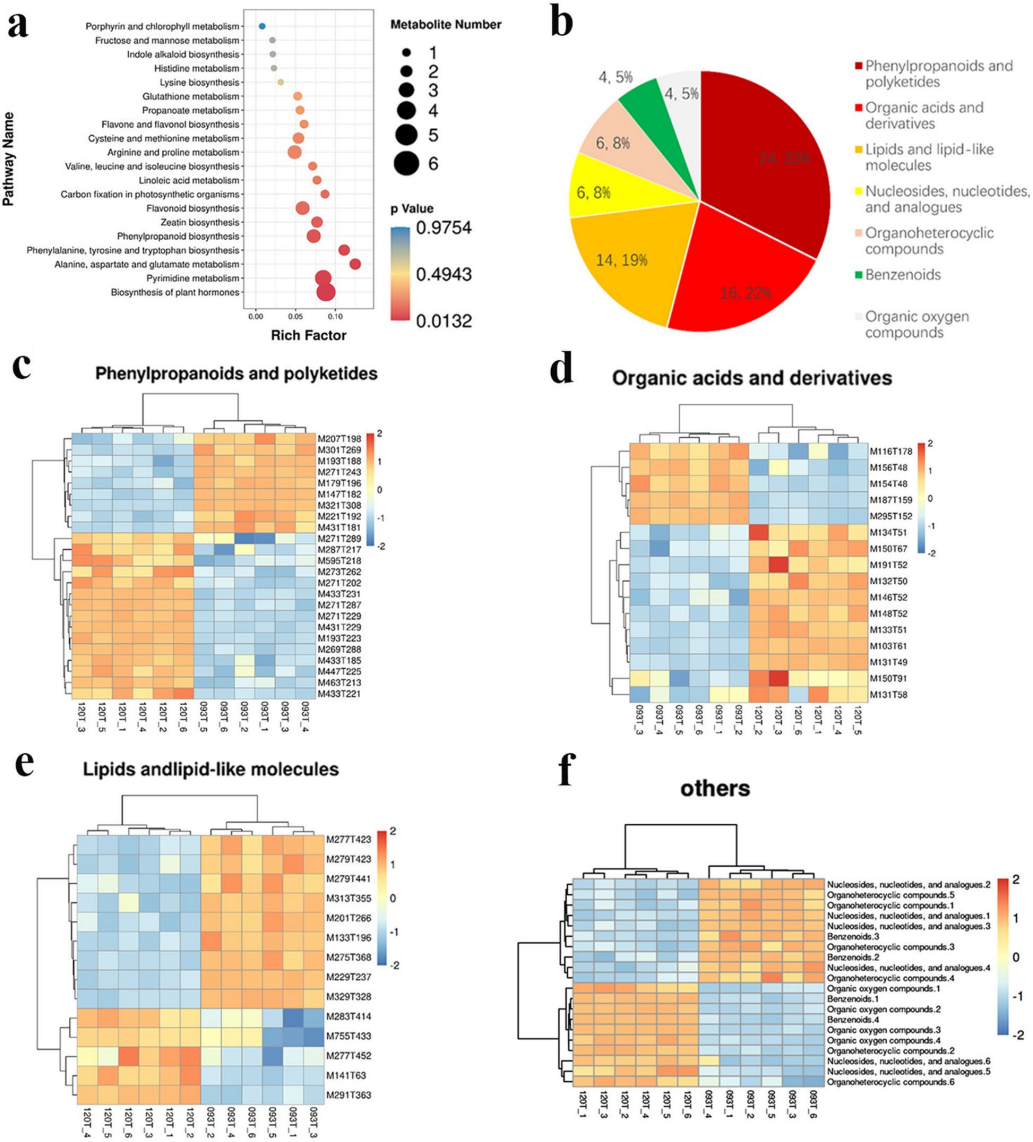
Comparison of metabolite levels between 093 and 120 under chilling stress. **a** Scatterplot of metabolites on the basis of KEGG. **b** The proportion of main metabolite species under chilling stress. **c–f** Three categories with a relatively high proportion are displayed in the **c**–**e** diagrams, and several categories with a relatively small proportion are uniformly displayed in the **f** diagram

**Fig. 5:**
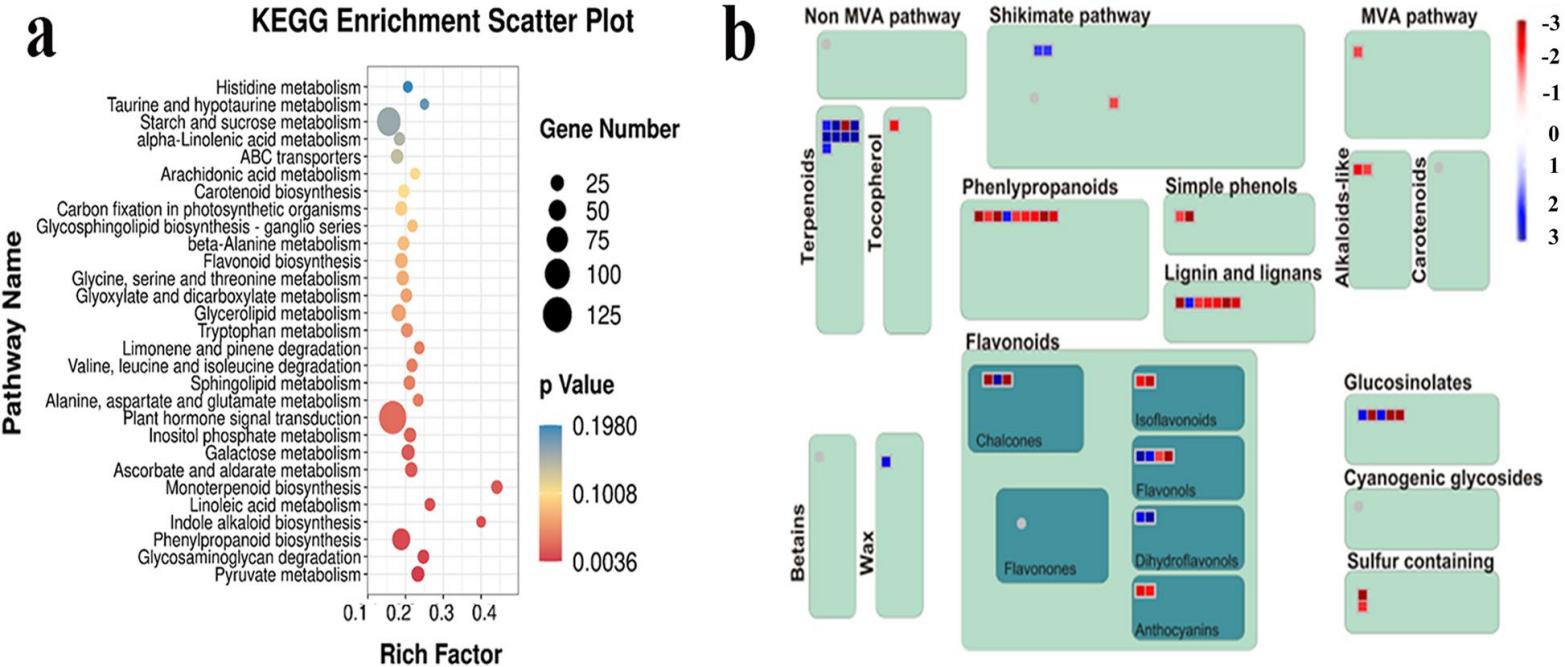
Comparison of gene levels between 093 and 120 under chilling stress. **a** The top 30 KEGG scatterplots under chilling stress.** b** MapMan was used to analyze 093CK and 120CK DEGs involved in secondary metabolism

A total of 102 DEMs (differentially expressed metabolites) were observed between the two genotypes under chilling stress. DEMs identified were analyzed using the KEGG pathway database. KEGG pathway analysis showed that “biosynthesis of plant hormones” was the most important subclass among various pathways, followed by “pyrimidine metabolism”. In addition, “biosynthesis of flavonoids” and various amino acid metabolic pathways showed significant enrichment (Fig. 4a). The 75 DEMs classified showed that phenylpropane and polyketones accounted for the highest proportion (24.33%), followed by organic acids and their derivatives (16.22%); lipids and their analogs (14.19%); nucleosides, nucleotides, and analogs (6.8%); and organic heterocyclic compounds (6.8%). The content of benzene ring compounds (4.5%) and organic oxygen compounds (4.5%) was the lowest (Fig. 4b). Most lipids and their analogs and organic acids and their derivatives were induced in 120 compared with 093(Fig. 4c and d), whereas most phenylpropane and polyketones were induced in 093 compared with 120 (Fig. 4e). In addition, most organic oxygen compounds were induced in 120; most nucleosides, nucleotides, and analogs, and organic heterocyclic compounds were induced in 093; and benzene ring compounds were induced in both 093 and 120 (Fig. 4f). To identify DEGs in the two contrast genotypes under chilling stress and compare the DEG expression level between them, 3141 DEGs were identified under chilling stress. The KEGG pathway analysis revealed that the most important subclass in each pathway was “starch and sucrose metabolism,” followed by “phenylpropane biosynthesis,” “flavonoid biosynthesis,” and “plant hormone signal transduction”. Furthermore, the metabolism of various amino acids changed (Fig. 5a and b).

### Comprehensive analysis of the metabolome and transcriptome

**Fig. 6:**
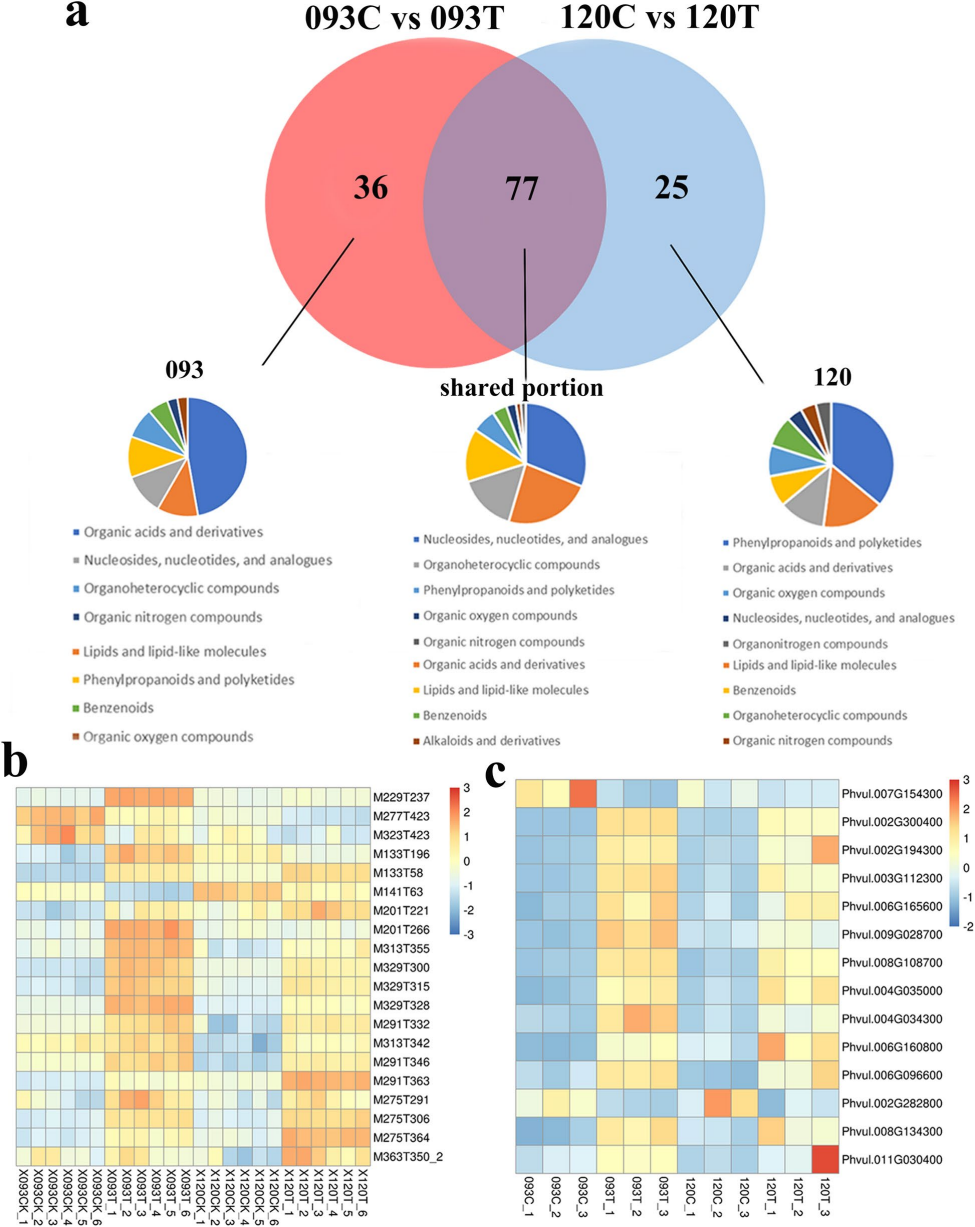
Comparison of metabolite and lipid levels between genotypes 093 and 120 under chilling stress. **a** Venn diagram of common (shared) metabolites between 093 and 120 under chilling stress, and classification of metabolites that accumulated only in 093 and 120. **b** Heat maps of lipid metabolites that respond to cold in 093 and 120. **c** Heat maps of lipid synthesis-related DEGs that respond to cold in 093 and 120

According to the Venn diagram, the total number of DEMs shared by 093 and 120 under chilling stress was 77, and most of them were specifically accumulated in the two genotypes: 093 had 36 DEMs,120 had 25 DEMs. Classification of common DEMs showed that organic acids and their derivatives were the metabolites most involved in the response of common bean to low temperature, followed by lipids and their derivatives. Only DEMs accumulated in 093 were divided into eight categories. The classification showed that phenylpropane and polyketones had the highest participation in low temperature stress (Fig. 6a). Under chilling stress, the accumulation of various lipids and their analogs increased significantly, but the accumulation was slightly lower in 093 than in 120 (Fig. 6b). In addition, the upregulation of expression levels of genes involved in lipid biosynthesis under chilling stress was higher in 093 than in 120 (Fig. 6c), indicating that the stress response of 093 under chilling stress was stronger than that of 120.

**Fig. 7:**
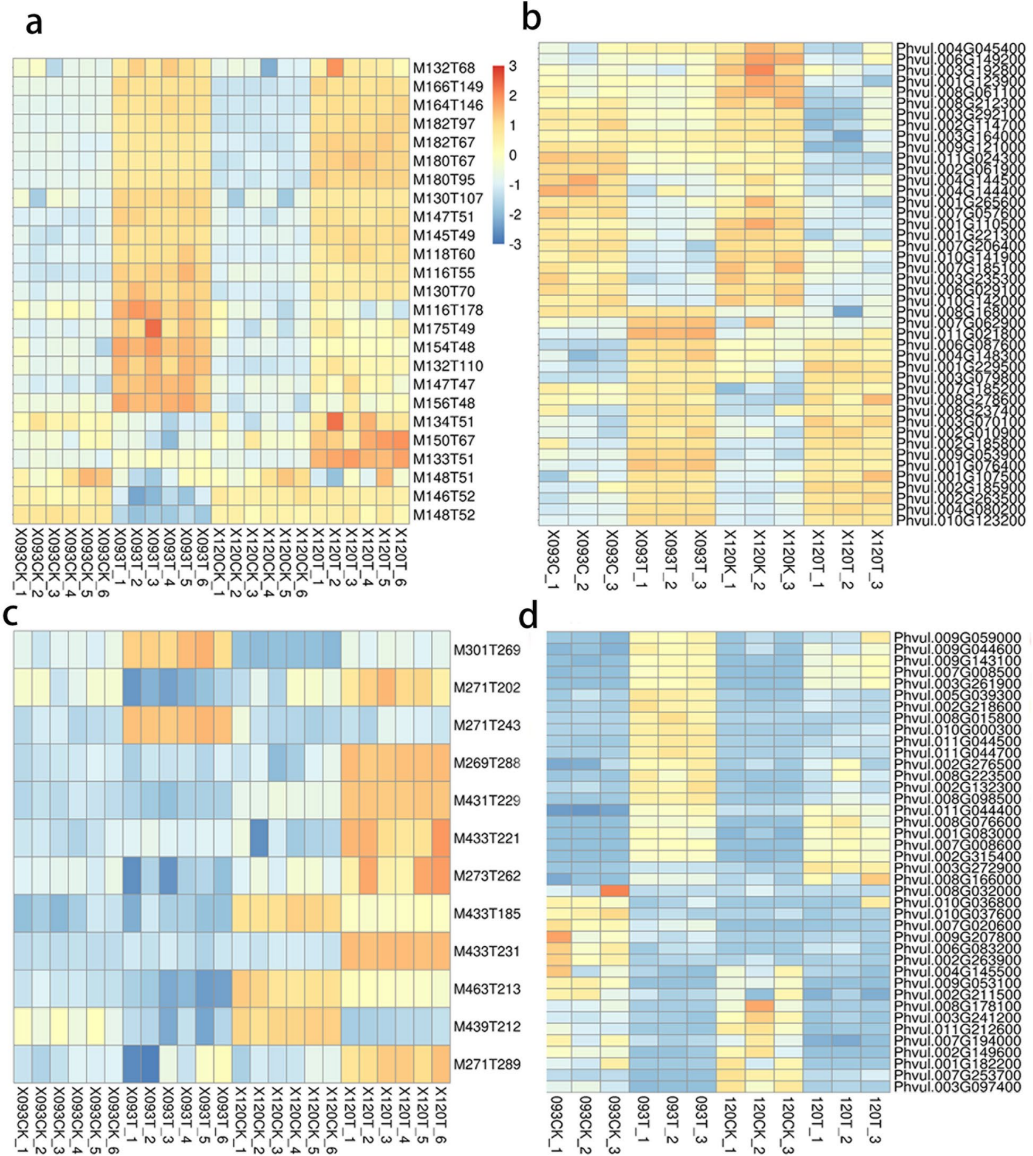
DEMs and DEGs involved in amino acid and flavonoid metabolism. **a** Amino acid metabolism-related DEMs. **b** Amino acid metabolism-related DEGs. **c** Flavonoid metabolism-related DEMs. **d** Flavonoid metabolism-related DEGs

Organic acids and their derivatives comprised the highest proportion of all types of amino acids(Fig. 6a). Heat map analysis of differences in amino acid accumulation showed that raised together amino acids have isoleucine (nonpolar), phenylalanine (nonpolar), polarity (neutral), leucine, tyrosine (nonpolar) and glutamine (neutral) polarity, and valine (nonpolar). The amino acids more enriched in 120 than in 093 were arginine (basic), histidine (basic), and lysine (basic), and amino acids enriched more in 120 than in 093 were aspartic acid (polar), methionine (neutral), asparagine (acidic), and glutamic acid (acidic). Under chilling stress, alkaline amino acids were enriched in 093 than in 120, whereas most acidic amino acids were enriched in 120 (Fig. 7a). Many DEGs involved in amino acid metabolism were also upregulated under chilling stress, and most genes in 093 and 120 had similar regulatory patterns (Fig. 7b).

Flavonoids and isoflavones are crucial components of phenylpropane and polyketone compounds (Fig. 6a), and the accumulation of most flavonoids was significantly higher in 120 than in 093 under chilling stress (Fig. 7c). Heat map analysis of DEGs showed that most genes in 093 and 120 had the same regulation mode, with a small number of genes having a different mode (Fig. 7d).

A total of 102 DEMs and 3141DEGs were observed between the two genotypes under chilling stress. Correlation analysis for differences in gene and metaboite expression was performed using the Pearson correlation coecient. A large number of related metabolites and related genes were identied in the 093 vs120. In the heat map of the correlation analysis, the horizontal axis represents genes, the vertical axis represents metabolites, red represents a positive correlation between genes and metabolites, and blue represents a negative correlation between genes and metabolites (Fig. 6b-c). Quercetin is a flavonoid compound with strong antioxidant capacity. Under chilling stress, quercetin was significantly accumulated in 093 and 120. Several flavonoids such as naringenin-7-O-glucoside were only upregulated in 120, and the differential expression of these flavonoids may be the reason for the different tolerance of 093 and 120 to chilling stress. In 093 and 120, the expression of a short-chain alcohol dehydrogenase (ABA2) (*phvul.005g031600*), which is a cytosolic short-chain dehydrogenase/reductase involved in the conversion of xanthoxin to ABA-aldehyde, was upregulated during ABA biosynthesis under chilling stress. In summary, the KEGG pathway analysis revealed that the flavonoid biosynthesis and endogenous hormones pathway was significant association with chilling stress.

### Differential gene expression verified by qRT-PCR

**Fig. 8:**
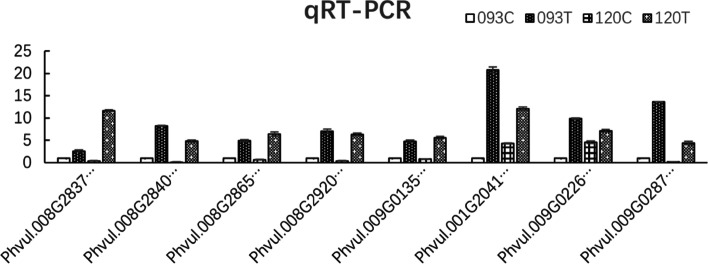
Under salt stress, eight genes upregulated in 093 and 120 were randomly selected from transcriptomic results for qRT-PCR analysis

Eight genes upregulated under chilling stress were randomly selected and verified by qRT-PCR. The gene expression map obtained by qRT-PCR was highly similar to that obtained using RNA-Seq data, confirming the reliability of RNA-Seq data (Fig. 8).

## Discussion

In northeast China, bean is one of the commonly used vegetable species with the highest nutritional content [27]. Because of the low latitude in northeast China, the growth of spring bean seedlings is easily affected by chilling injury, which severely limits bean production. And will affect the grain quality and composition of edible beans [28]. Low temperature tolerance of plants is a quantitative trait, and the transcription, translation and post-translational modification of genes will change after chilling stress. There are many studies on chilling stress in Arabidopsis thaliana, a model crop, and some reaction pathways involved in chilling stress, such as CBF pathway, have been found [29]. However, not all crops have the same way to resist chilling stress. For example, CBF has a relatively small role in tomatoes [30], rice [31] and corn [32]. Therefore, it is generally believed that different types of crops have different responses to chilling. The study on the chilling tolerance of falcate alfalfa in leguminous plants showed that plant hormone signal transduction, endoplasmic reticulum protein processing, carbon metabolism, glycolysis/gluconeogenesis, starch and sucrose metabolism, and endocytosis pathway were all differentially expressed during its response to low temperature [33]. Sucrose starch metabolism in chickpea is related to abiotic stress tolerance or sensitivity of plants. Under LT (low temperature) conditions, changes in sugar and starch content in chickpea are found, and amylase related to cold tolerance mechanism of chickpea is found [34]. We investigated the resistance mechanism of common bean to chilling stress by comparing the cold tolerance of early type variety 120 with the that of the cold-sensitive variety 093.

**Fig. 9:**
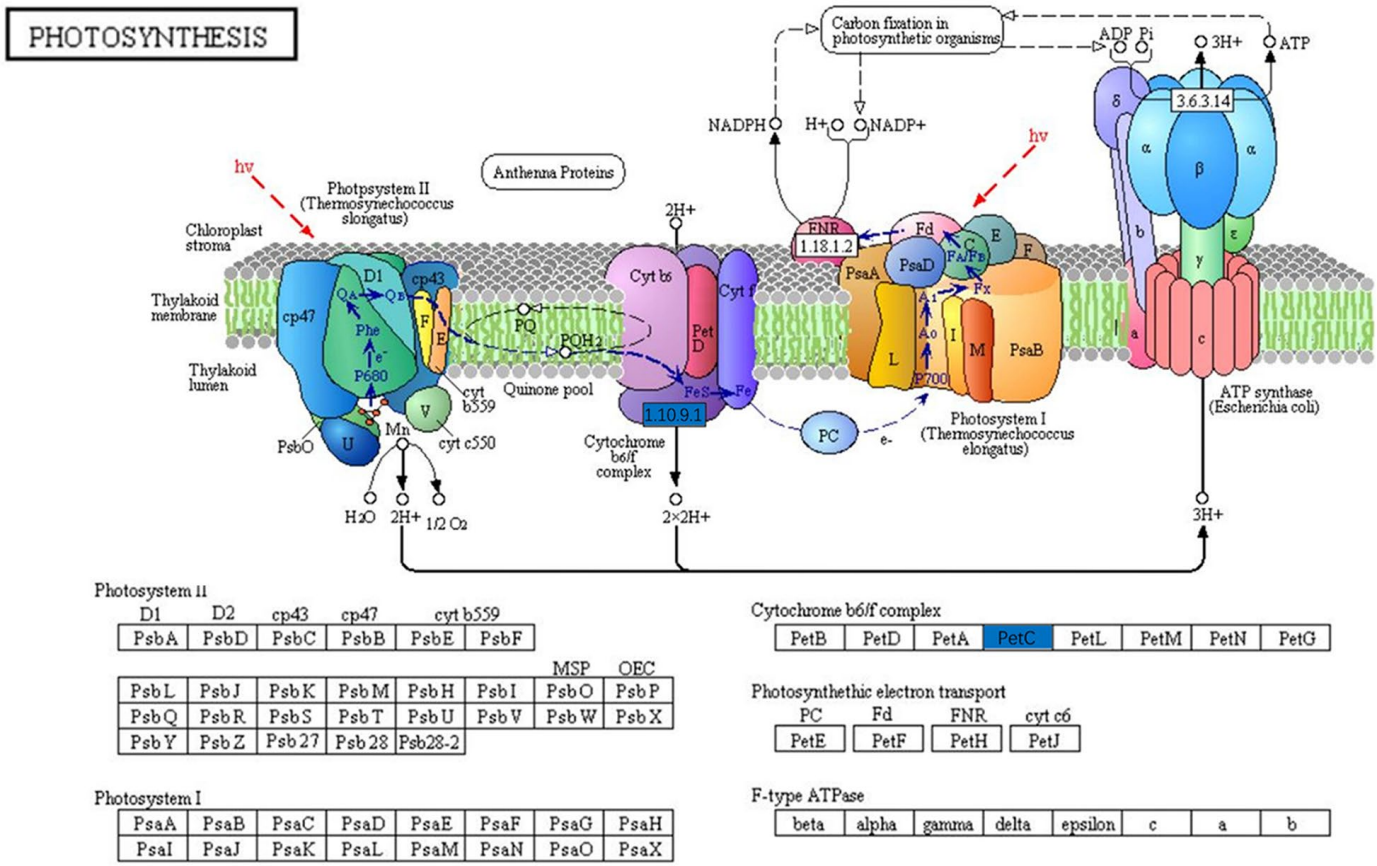
Chematic diagram of photosynthesis; *petC* is shown in blue

Photosynthetic indices of 093 were greater under non-chilling stress, but the indices of the two varieties declined to about the same level under chilling stress. The decrease in photosynthetic indices could be linked to biofilm system damage caused by low temperature, which reduces the flexibility of the cell membrane, impacting photosynthetic reactions on the thylakoid membrane [35]. MDA, a result of membrane lipid peroxidation, can be used to assess cell membrane damage [36]. Under chilling stress, the MDA content of 093 increased significantly, while that of 120 was almost changed, which was verified by the fact that the photosynthetic index of 093 changed more dramatically. Transcriptome results showed that Rieske FeS protein (*petC*) expression of the b6f complex was downregulated in both 093 and 120 photosynthetic systems under chilling stress. Studies have shown that *petC* of the Arabidopsis thaliana cytochromic b6f complex was overexpressed. The *petC* gene can increase electron transfer efficiency [37], whereas its deletion leads to the blockage of electron flow, and eventually plant death [38]. The change in the *petC* expression level may be one of the reasons leading to the decline in photosynthetic indices (Fig. 9).

In addition to the common reactions of lipids to cold, colnelenic acid (CnA) accumulation was found in 093 and 120 under chilling stress. Some studies have shown that CnA accumulates in potato leaves after fungal infection, indicating that it plays a major role in potato resistance to fungal infection [39]. CnA is also believed to play a role in chilling stress resistance in common bean, but additional studies are warranted to confirm this belief. In addition to accumulation of lipids and their derivatives, the accumulation of different amino acids was dramatically upregulated during chilling stress. Moreover, alkaline amino acids were found to be more enriched in 093 than in 120, while most acidic amino acids were enriched in 120. Researchers have not focused on different acidic and basic amino acid buildup in 093 and 120. Proline is essential for plant resilience to chilling stress because it plays various roles such as an osmotic regulator, a protein and plasma membrane stabilizer, an osmotic pressure-related gene inducer, a reactive oxygen scavenger, and a stable source of nitrogen and carbon during late recovery growth [40]. In this experiment, proline content in 093 and 120 increased significantly under chilling stress. Lipid peroxide damage in primary and secondary lysosome membranes, causing the body to dissolve enzyme containing released as hydrolysis acid phosphatase, to cells and mitochondrial membrane damage important organelles, methionine through various ways to Fight the damage [41]. Under chilling stress, methionine content in 120 was clearly enhanced, whereas that in 093 was not enriched. One of the reasons for the differences in chilling stress tolerance between 093 and 120 could be differential methionine enrichment.

Flavonoids, a class of secondary plant phenols, have significant antioxidant and chelating properties [42]. The protective effects of most flavonoids in biological systems are attributable to their ability to transfer electrons, scavenge free radicals, and chelate metals[43] to activate antioxidant enzymes [44], reduce α-tocopherol free radicals [45], and inhibit oxidases, respectively. In particular, flavonoids play a defensive role in abiotic stress. Quercetin is a flavonoid compound with strong antioxidant capacity. Under chilling stress, quercetin was significantly accumulated in 093 and 120. Several flavonoids such as naringenin-7-O-glucoside were only upregulated in 120, and the differential expression of these flavonoids may be the reason for the different tolerance of 093 and 120 to chilling stress. Enrichment analysis of DEGs showed that several flavonoid metabolism-related genes were upregulated under chilling stress, such as dihydroflavanol oxidreductase (phvul.009G04460), chalone isomerase (phvul.009G143100), and flavonoid 3-hydroxylase (phvul.003G261900) (Fig. 7d).

**Fig. 10:**
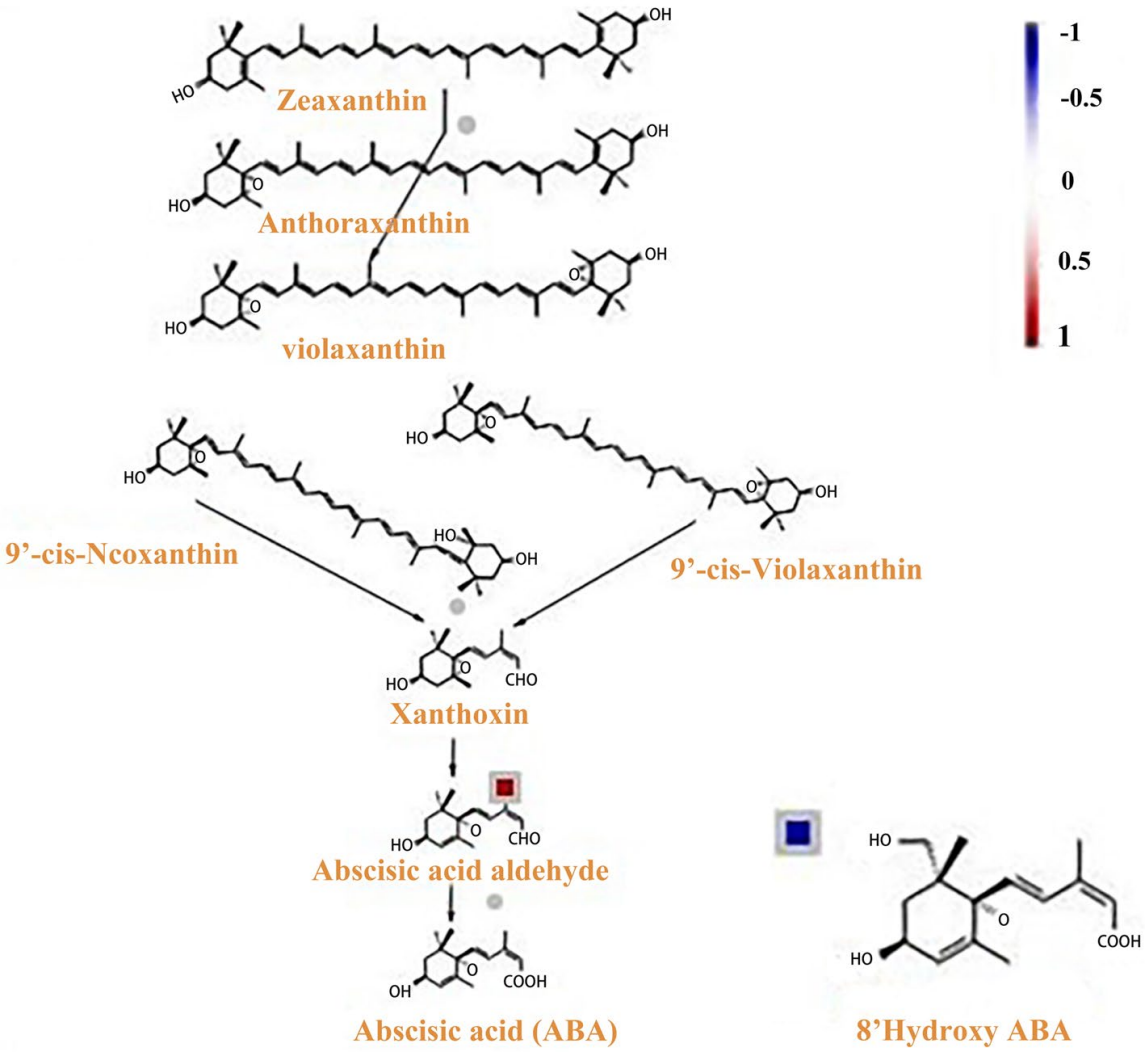
Under chilling stress, the ABA biosynthesis pathways of 093 and 120 were upregulated (red) and downregulated (blue), respectively

**Fig. 11:**
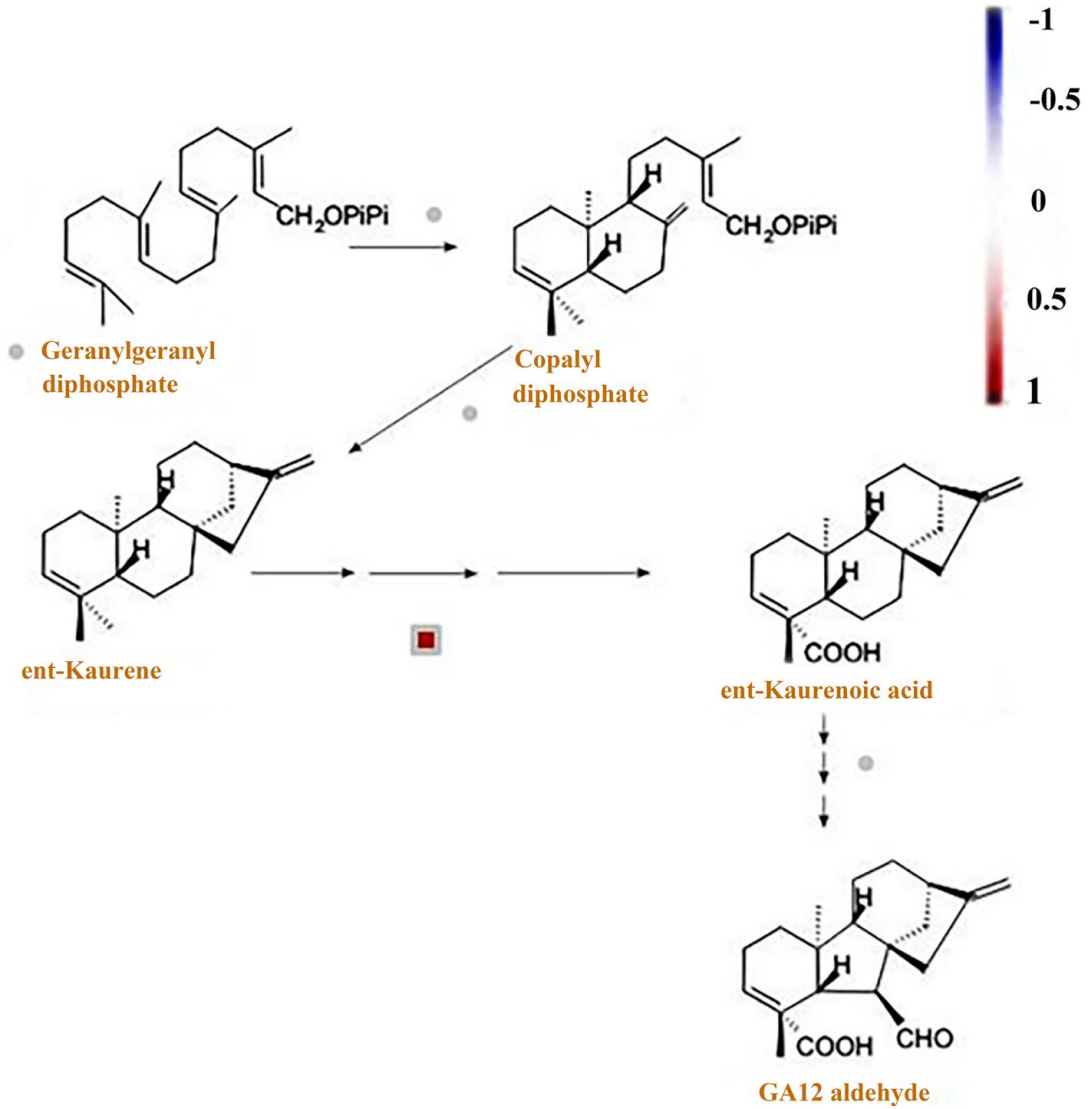
A biosynthesis pathways in 093 and 120 under chilling stress

**Fig. 12:**
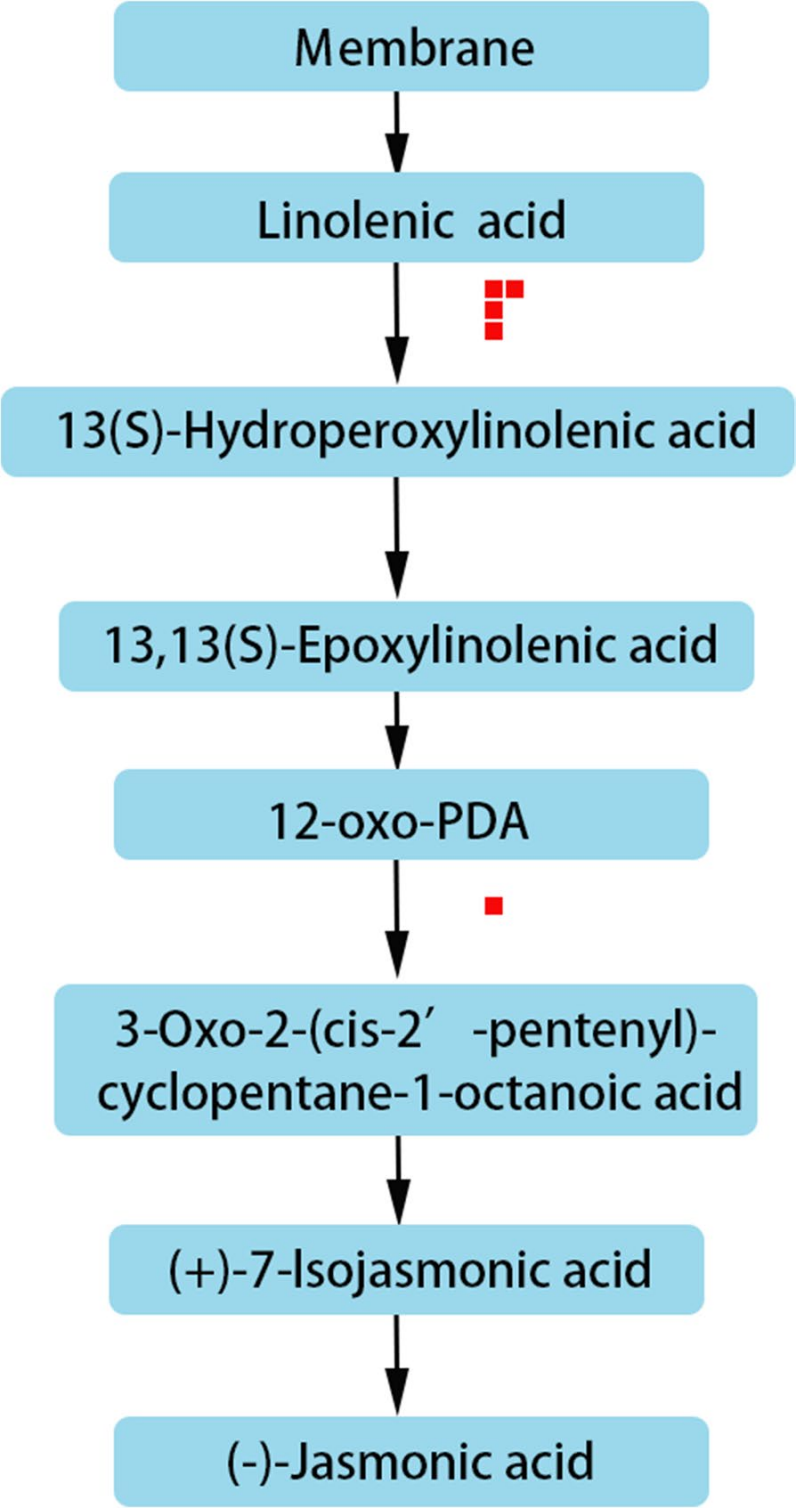
A synthesis pathways in 093 and 120 under chilling stress

In the chilling stress response, plant endogenous hormones change. showed that after 1 day of low temperature domestication, ABA content in winter wheat increased rapidly, the cytokinin level with biological activity decreased, the GA level with abiotic activity increased, and the content of the ethylene precursor amino cyclopropanecarboxylic acid increased. After 3–7 days of acclimation, the content of ABA, cytokinin, and GA decreased, whereas the content of salicylic acid and JA began to increase [46]. After hormone treatment, tea plant [47] and *A. thaliana* [48] exhibited enhanced low temperature tolerance. Studies have shown that ABA plays a key role in plant response to chilling stress [49–51]. In 093 and 120, the expression of a short-chain alcohol dehydrogenase (ABA_2_) (phvul.005g031600), which is a cytosolic short-chain dehydrogenase/reductase involved in the conversion of xanthoxin to ABA-aldehyde, was upregulated during ABA biosynthesis under chilling stress. Moreover, the expression of 8-hydroxylase (phvul.003g278400), which is a protein with ABA 8'-hydroxylase activity and is involved in ABA catabolism, was downregulated during chilling stress. This indicated that compared with 093, ABA anabolism in 120 was inhibited and ABA catabolism was promoted (Fig. 10), thereby keeping the ABA content in 120 lower. We also found that the expression level of ent-Kaurene oxidase, which is involved in GA metabolism, was higher in 093 (Fig. 11). This protein is a member of the CYP701A cytochrome p450 family that is involved in later steps of the GA biosynthesis pathway. JAs are synthesized from αlinolenic acid in the chloroplast membrane as a substrate [52]. Several genes in the JA biosynthesis pathway were differentially expressed in 093 and 120 (phvul.010G135800, phvul.005G156900, phvul.007g055600, phvul.002G228700, phvul.002G043000). The expression levels of these genes were all higher in 093 than in 120 (Fig. 12). The differential expression of different hormones in 093 and 120 may affect the tolerance of common bean to low temperature stress.

## Conclusions

**Fig. 13:**
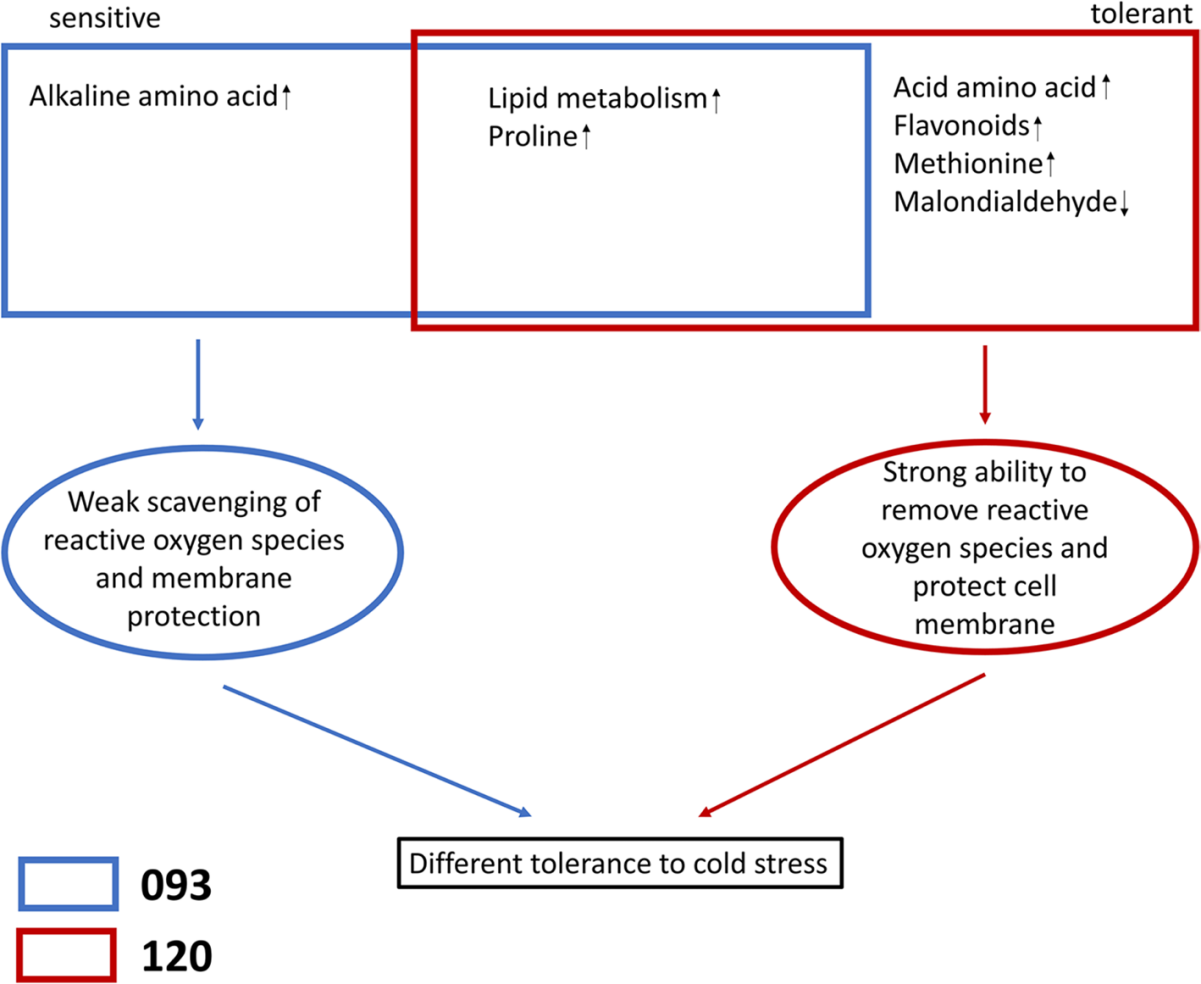
Under chilling stress, lipid metabolism, and proline, acid amino acid, and methionine content were significantly upregulated, and MDA was significantly downregulated in 120 compared with 093, which gave 120 a stronger ability to scavenge reactive oxygen species and protect the cell membrane. However, only lipid metabolism, and basic amino acid and proline content were upregulated in 093, and its scavenging and protection abilities were weak

In this study, using metabolome and transcriptome analyses, DEGs and DEMs in the cold-sensitive cultivar 093 and the cold-tolerant cultivar 120 were evaluated following cold stress treatment. The transcriptome and metabolome data suggested that 093 and 120 have similar cold stress responses, while some differences do exist. For example, the acid amino acid, flavonoids, methionine content of 120 increased malondialdehyde content of 120 decreased after cold stress (Fig. 13), which may be the reason for the difference in cold tolerance between 093 and 120. In addition, the response of common beans to cold stress does not depend on CBF pathway, and the expression levels of ABA, GA and JA and the changes of various substances in ROS system may play a more important role. Flavonoids, methionine and malondialdehyde can be used as cold stress markers in the identification of cold tolerance of common bean seedlings, and hormones can be applied to help common bean seedlings resist cold stress. This study provides a preliminary explanation for the molecular mechanism and response pathway of common bean in response to cold stress, and also provides a basis for integrating cold tolerance genes to improve the cold tolerance of common bean in the future.

## Data Availability

Transcriptome data have been uploaded to the GEO database: https://www.ncbi.nlm.nih.Gov/geo/, Registration number is: GSE192891.
